# Rat perivascular adipose tissue microvasculature revealed by tissue clearing

**DOI:** 10.3389/fphys.2024.1535711

**Published:** 2025-01-31

**Authors:** Stephanie W. Watts, Emma Flood, Brian D. Gulbransen, William F. Jackson

**Affiliations:** ^1^ Department of Pharmacology and Toxicology, Michigan State University, East Lansing, MI, United States; ^2^ Department of Physiology, Michigan State University, East Lansing, MI, United States

**Keywords:** perivascular adipose tissue, tissue clearing, EZClear, lectin, microvasculature, rat

## Abstract

Perivascular adipose tissue (PVAT) surrounds the majority of blood vessels and plays diverse roles in vascular and metabolic functions. The hormonal and lipid exchange functions of PVAT require access to blood vessels. However, the microvascular supply of PVAT, especially in rats, remains enigmatic due to technical restraints involved in imaging fat depots. Therefore, we developed and validated an approach to visualize the microvasculature of PVAT in rats. In this study, we report a stepwise protocol as a method to clear and visualize the microvasculature of the thoracic aortic PVAT in the Dahl salt-sensitive (SS) rat. Blood vessels are first traced in anesthetized rats using *Lycopersicon esculentum* (tomato) lectin DyLight 649 (Lectin 649). The dissected aorta with intact PVAT is then subjected to a stepwise clearing protocol over 12 days, followed by imaging on a Nikon confocal microscope. Images were stitched together to visualize cross sections of the whole vessels. The microvasculature of aortic PVAT is present and profoundly dense, and it is similar in the ventral and lateral lobes of aortic PVAT. This developed method is adoptable and adaptable to other PVATs in rats.

## 1 Introduction

Our laboratories are committed to unveiling new functions of perivascular adipose tissue (PVAT). Rediscovered by Soltis and Cassis (1991), this often overlooked part of the vasculature has become a subject of great interest because of its diverse functions and its contributions to the health or dysfunction of arteries in disease (Aghamohammadzadeh et al., 2011; Britton and Fox, 2011; Fernandez-Alfonso et al., 2017; Horimatsu et al., 2017; Lehman et al., 2010; Nosalki and Guzik, 2017). We recently performed a single-nuclei RNA sequencing experiment that revealed the presence of smooth muscle and endothelial cells in the PVAT that surrounds the thoracic aorta of the rat (Dahl SS strain) (Thompson et al., 2024). PVAT is the fat that *surrounds* the vessel. The presence of smooth muscle and significant endothelial cell clusters raised the question of the genesis of these cells. We considered that, like other brown and white fats (Crandall et al., 1997; Xue et al., 2010), PVAT must have its own microvasculature.

We developed a method for clearing the rat thoracic aortic PVAT, recognized as a type of brown fat (Fitzgibbons et al., 2011; Hildebrand et al., 2018; Padilla et al., 2013) for the visualization of its microvasculature using injected lectin. This was based on previous work in mice, which have significantly less dense tissue (Hanscom et al., 2024), and was adapted from a number of protocols for clearing adipose tissue (Chi et al., 2018; Lin et al., 2022; Luong et al., 2021). The formally recognized brown adipose tissue (BAT), located subscapularly, has a microvasculature identified by polymer dots (Li et al., 2023), microvasculature identified by (immuno)fluorescence (Li et al., 2019; Nnodim and Lever, 1988), a measurable blood volume (Ron et al., 2019), and a microvasculature that can undergo rarefaction in obesity (Shimizu et al., 2014). Therefore, there are *bona fide* scientific reasons to hypothesize that the brown fat surrounding the thoracic aorta would also possess microvasculature. Although one goal of the present work is to test this hypothesis, a greater goal is to provide a detailed method/protocol that guides an experiment from the preparation of the rat PVAT through clearing to the analysis of generated images of thoracic aortic PVAT microvasculature.

This protocol is useful in a few ways. First, this has not been applied to tissues from rats before. Second, thoracic aortic PVAT, as a type of brown fat, presents a greater challenge than white adipose tissue because of its inherent color. Third, thoracic aortic PVAT is also one of the densest PVATs surrounding rat vessels, necessitating a whole-animal approach rather than incubating the fat in lectin (sufficient penetration of PVAT does not occur with *in vitro* incubation with lectin). Technically, our success in this study supports the application of this developed protocol to other rat vascular beds. Scientifically, we discovered that PVAT has a profoundly rich microvasculature, which raises many questions about the health of the PVAT microvasculature in disease and its contributions in health.

## 2 Procedure and materials

### 2.1 Procedure

**FIGURE 1:**
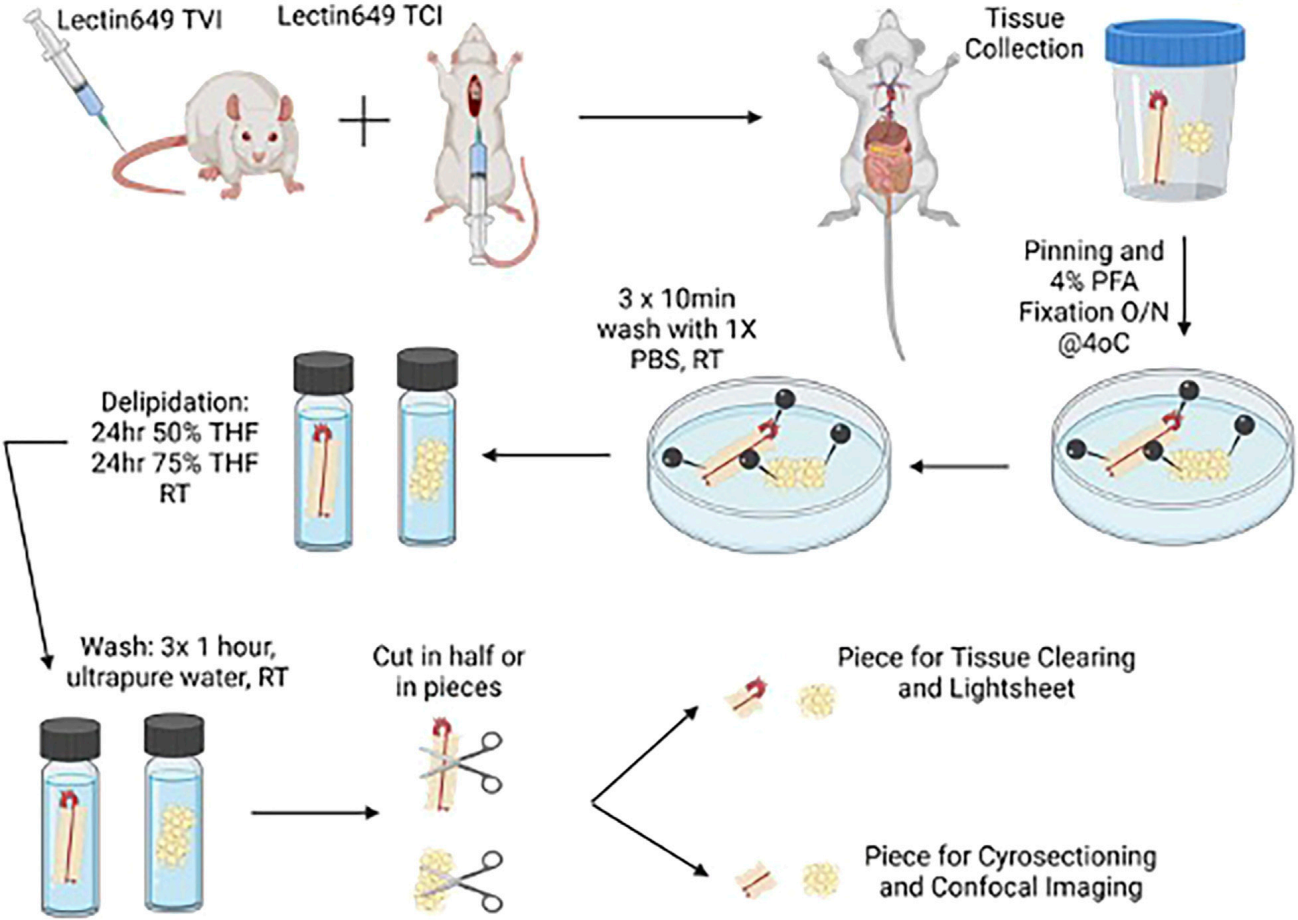
Cartoon depicting injection of lectin in rats for the removal of tissues for clearing. Created with BioRender. Alternative paths of study (light sheet microscopy) are depicted in this figure.

Figure 1 depicts the overall steps described from animal injection to aortic dissection.

#### 2.1.1 Day 1: Lectin 649 injection, tissue collection, and fixation

##### 2.1.1.1 Lectin 649 and tissue collection

Labeling of the vasculature in rats was carried out via a lateral tail vein injection of Lectin 649. In brief, rats (three male Dahl SS rats, ∼300 g, Charles River Laboratories, on a normal diet) were anesthetized in an induction chamber using 3% isoflurane delivered in 100% O_2_. Once a surgical plane of anesthesia was reached, the rats were transferred to a nose cone supplied with 2% isoflurane in 100% O_2_ and positioned on their side to allow easy access to the lateral tail vein. The absence of hind paw reflex was confirmed before proceeding. After cleaning the tail with a sterile ethanol pad, a 31G insulin needle was used to inject 500 μL of Lectin 649 into the lateral tail vein. The needle was gently inserted into the vein but not passed through it. Correct needle placement within the tail vein was confirmed by withdrawing the plunger to observe a small amount of blood flow into the syringe prior to lectin 649 injection, as well as lack of resistance when injecting Lectin 649 into the vein. Lectin was injected into the tail vein over a period of 60 s. Following the lectin injection, the rats were removed from anesthesia and returned to their home cages for 30 min to allow for the circulation of the injected lectin.

After these 30 min, anesthesia was reinduced with 3% isoflurane/100% O_2_ induction in an induction chamber and 2% isoflurane/100% O_2_ maintenance in a nose cone. After positioning the rat supine and checking for the loss of hind paw reflex, the abdominal wall was cut to expose the diaphragm and the end of the sternum. Blunt forceps were used to grip the end of the sternum/xiphoid process to pull the sternum/diaphragm back toward the heart, rendering the heart visible through the diaphragm. An additional bolus of Lectin 649 (150 μL) was injected over 60 s directly into the left ventricle through the diaphragm using a 31 G insulin needle. The needle was kept in place for an additional 60 s to ensure that Lectin 649 was pumped out of the chamber and did not leak from the heart. The hold on the sternum was released at this point. Another 5 min of anesthesia was allowed for further circulation of this bolus of lectin, with continual confirmation of the lack of hindpaw reflex response. Once these 5 min were completed, the rib cage on both sides was dissected to expose the heart. A small incision was made in the right atrium, and a 19 G needle with an attached syringe prefilled with sterile 0.9% saline was inserted into the left ventricle for transcardial perfusion. Saline (50 mL) was introduced into the left ventricle at a rate of ∼10–13 mL/min. Successful perfusion was confirmed by the color change of the kidneys and liver, transitioning from dark red to light pinkish/beige color. Once saline perfusion was complete, the thoracic aorta, beginning just above the diaphragm and with the heart attached, was removed and placed into a specimen cup containing ice-cold DMEM/F12. Tissues were kept on ice in a light-protected container until ready to fix all collected tissues.

##### 2.1.1.2 Tissue fixation

**FIGURE 2:**
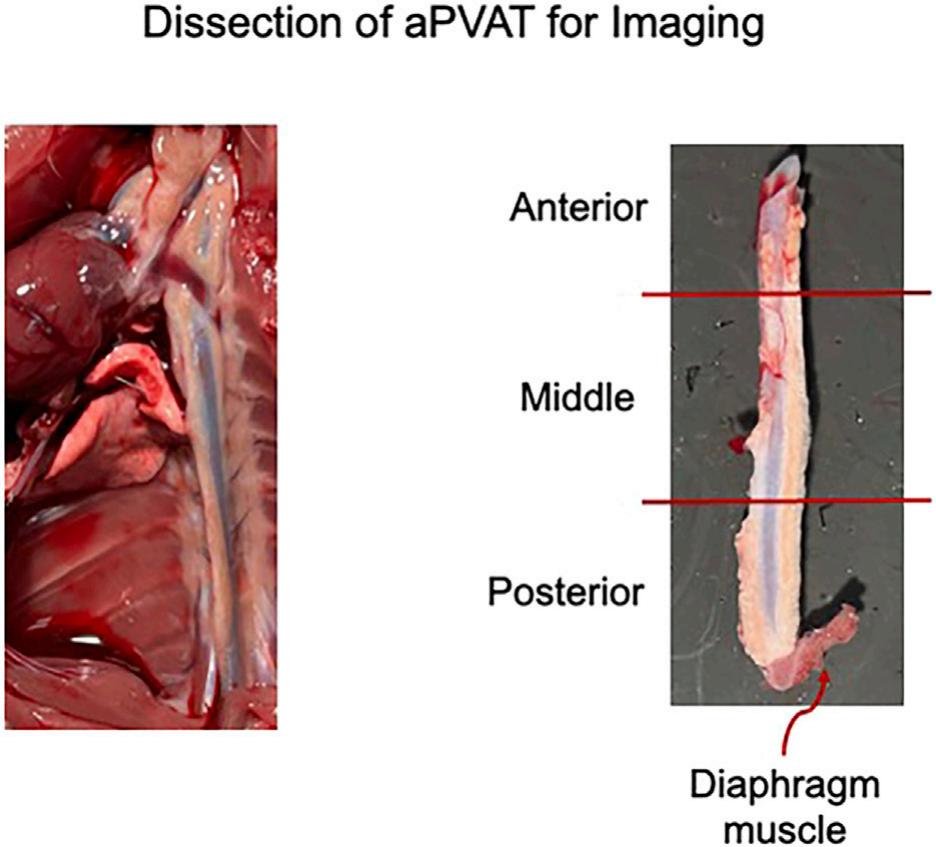
Left: representative image of the thoracic aorta and heart *in situ* post lectin infusion. Right: sections of the isolated thoracic aorta divided into posterior, middle, and anterior segments. Representative of the three male rats used in this study.

The collected tissue was pinned out in a Sylgard-filled dish in a linear orientation, extending from the heart to the bottom of the thoracic aorta, including the diaphragmatic tissue (Figure 2); this was performed in ice-cold DMEM/F12. DMEM/F12 was then removed and replaced with 4% paraformaldehyde (PFA) in the dish. Tissues were fixed overnight at 4°C in plastic containers covered with foil to protect them from light.

#### 2.1.2 Day 2: initial tissue delipidation

The fixed tissue was removed from the covered plastic containers stored at 4°C and washed three times for 10 min each with 1× PBS at room temperature on an orbital rocker, with samples protected from light. The fixed, pinned tissue was transferred to a Petri dish containing 1× PBS and cut into smaller pieces (anterior, middle, and posterior thoracic aPVAT; Figure 2, right) using a scalpel. Tissues not immediately used were stored at 4°C in 5-mL glass scintillation vials filled with 1× PBS and placed in plastic containers covered with foil to protect them from light. We followed the EZClear protocol with minor modifications (Hsu et al., 2022). A 50% tetrahydrofuran (THF) solution was prepared by mixing equal parts ultrapure water with 100% THF for tissue delipidation. A measure of 10 mL of 50% THF was added to 10-mL glass scintillation vials, and one tissue piece was added to each vial. The scintillation vials were capped, lid-secured with parafilm to ensure no leakage, and placed in a secondary container lined with paper towels to help keep the vials upright. The secondary container was wrapped in a foil to protect the tissue from light. The tissue was then delipidated for 24 h at room temperature while rocking on an orbital shaker in a fume hood.

#### 2.1.3 Day 3: continuation of tissue delipidation

As a modification to the EZClear protocol, delipidation was continued for an additional 24 h by removing 10 mL of 50% THF from the vials and replacing with 10 mL of 75% THF. The 75% THF solution was prepared by adding 7.5 mL of 100% THF to 2.5 mL of ultrapure water. The lids were again secured with a parafilm, and the vials were placed back into the secondary container, which was covered to protect the tissue from light. This tissue was delipidated at room temperature, rocking on an orbital shaker in a fume hood, for an additional 24 h.

#### 2.1.4 Day 4: tissue wash and 10% sucrose gradient start

The 75% THF solution was removed from the delipidated tissue, and the tissue was washed three times for 1 h at room temperature with 10 mL of ultrapure water. The vials containing the tissue and water were placed back into the secondary container and covered with foil during the washing steps to protect the tissue from light.

**FIGURE 3:**
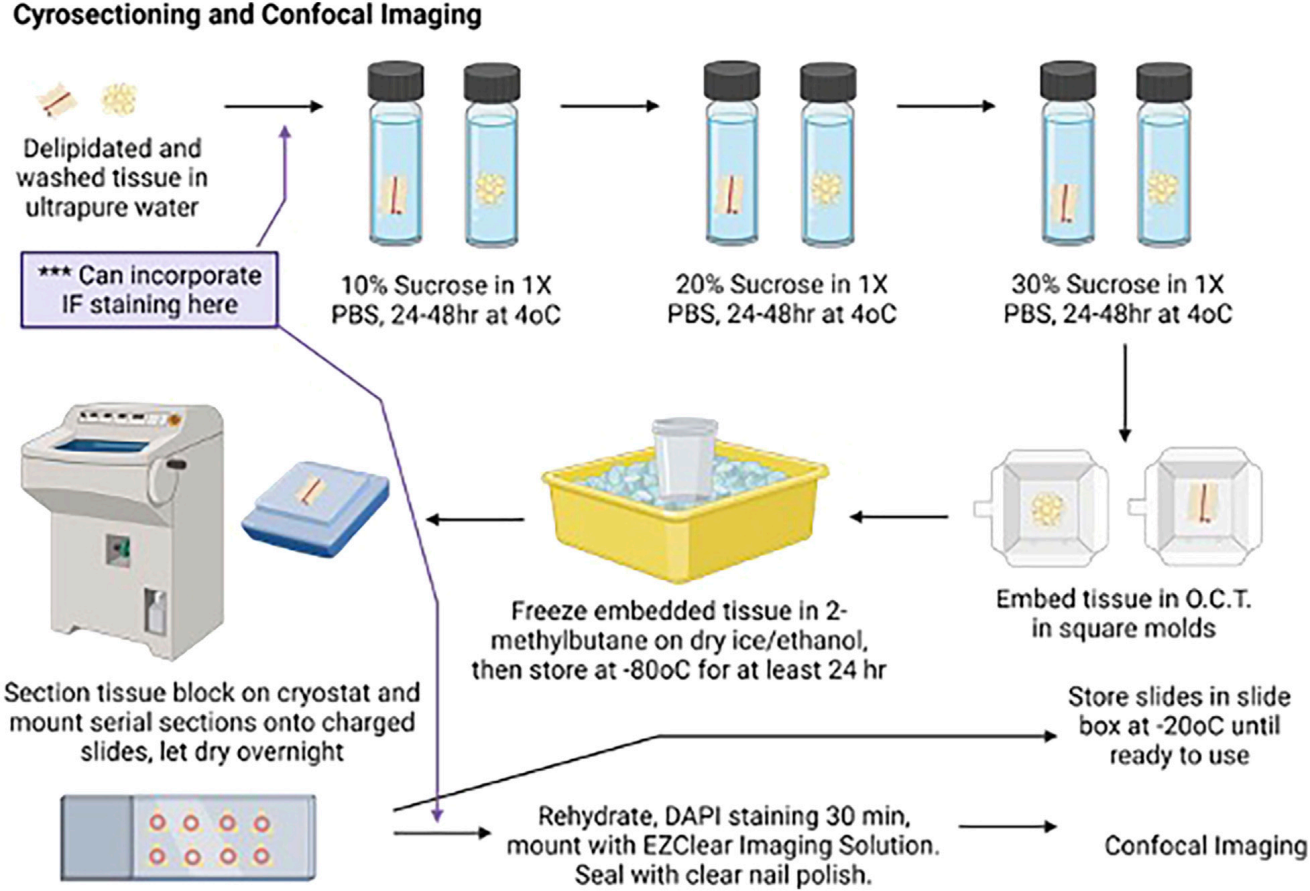
Cartoon depicting aortic ring delipidation to confocal imaging. Created with BioRender. An alternative path for the immunofluorescence study is depicted here.

While the wash steps were ongoing, sucrose gradients were prepared for the cryoprotection steps. A measure of 10%, 20%, and 30% sucrose solutions were prepared by adding 5 g, 10 g, or 15 g sucrose to 250 mL of 1× PBS, respectively, and then filter-sterilizing the solutions. Once wash steps were completed, the delipidated tissues were transferred to 15-mL centrifuge tubes containing 14 mL of 10% sucrose/1X PBS. The tubes were covered with a foil and stored upright at 4°C for 48 h (first step in Figure 3).

#### 2.1.5 Day 5–6: 20% sucrose gradient start

The 10% sucrose solution was replaced with 20% sucrose/1× PBS, and the delipidated tissues remained in the tubes, covered with a foil to protect them from light. The tubes were left upright at 4°C for 48 h.

#### 2.1.6 Day 7–8: 30% sucrose gradient start

The 20% sucrose solution was replaced with fresh 30% sucrose; the tubes were covered with a foil to protect them from light and left upright at 4°C until ready for embedding and cryosectioning.

#### 2.1.7 Day 8: tissue embedding in O.C.T

Tubes containing cryoprotected tissues were removed from 4°C and warmed to room temperature. The tissue piece was removed from the 15-mL tube with curved forceps using a scooping method to prevent tissue damage, transferred to a Kimwipe, and gently rolled to remove excess sucrose. The tissue was then transferred to a square embedding mold using the curved forceps and fully covered with O.C.T. The tissue and O.C.T. were frozen in a specimen cup containing 2-methylbutane placed in dry ice and 100% ethanol. After the O.C.T. was frozen, the mold was removed from 2-methylbutane, wrapped in foil, and stored on dry ice in a covered container (Figure 3). After all tissues were embedded and frozen, the molds were transferred to a plastic bag and stored at −80°C until sectioning, for no less than 24 h after being placed at −80°C.

#### 2.1.8 Day 9: sectioning on cryostat

The embedded tissue was removed from the −80°C plastic bag and placed in a cryostat chamber to warm up; the chamber was maintained at −20°C for 30 min prior to sectioning. Embedded blocks were positioned on the mounting pedestal with O.C.T., ensuring that the subsequent serial sections were cut perpendicular to the long axis of the aorta. This orientation ensured that yielded sections contained the aorta surrounded by all three lobes of aPVAT. Tissue was sectioned at 60 µm, with serial sections being collected and directly mounted onto charged microscope slides in order to enable digital imaging and stitching of the microvasculature. Careful mounting of serial sections ensured the continuity of orientation for later analyses. All slides were placed in an opaque slide tray to protect them from light and left to dry overnight at room temperature in a closed drawer, protected from light.

#### 2.1.9 Day 10: DAPI staining, clearing, and mounting coverslips for confocal imaging

**FIGURE 4:**
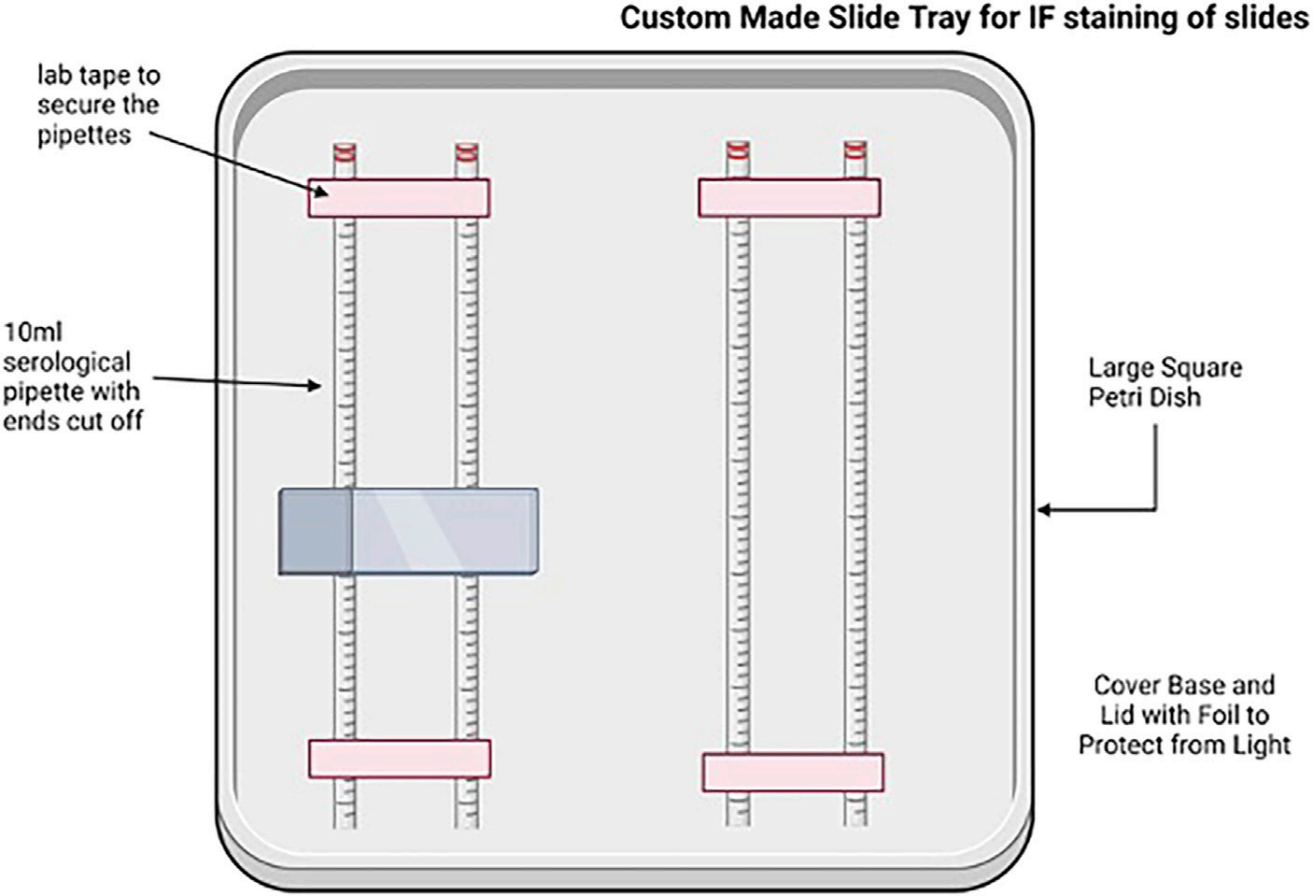
Cartoon depicting the homemade tray for the immunofluorescence staining of sectioned tissues. Created with BioRender.

One slide with 3–6 sequential sections for each tissue piece was selected for imaging. The remaining slides were transferred from the slide tray to a slide box and stored at −20°C. A pap pen was used to delineate the outer boundaries of each slide, and the slides were allowed to dry at room temperature in a handmade tray (Figure 4), protected from light. Once the hydrophobic barrier dried, slides were rehydrated with 1× PBS for 10 min at room temperature on the tray, protected from light. After 10 min, the 1× PBS was removed from the slides and replaced with the DAPI solution (1:5,000 dilution of 1 mg/mL stock in 1X PBS) to label the nuclei. Slides were incubated with the DAPI solution for 60 min in the tray at room temperature, protected from light. After 60 min, the DAPI solution was removed, and the slides were washed twice with 1× PBS for 5 min at room temperature, protected from light. A final rinse with ultrapure water was performed, and then, the EZClear Imaging Solution (200 μL per slide) was added along the bottom of the slides, avoiding air bubbles. The coverslip was positioned over the slide along the bottom edge and gently lowered onto the slide from the bottom up to facilitate the spread of the EZClear Imaging Solution upward as the slide was lowered, covering all sections fully (Figure 3). The slide was set aside in another identical handmade tray, protected from light, and allowed to set for a few minutes. Slides were then sealed along the edges of the coverslip with clear nail polish. The sealed slides were allowed to set overnight at room temperature, protected from light in the trays, and then stored in slide boxes at room temperature until ready for imaging.

#### 2.1.10 Day 11 and on: imaging

Large area scans of 3–6 sequential sections were acquired on a Nikon AXR Confocal Microscope equipped with a Nikon 25X CFI75 APO LWD water immersion objective (1.1 numerical aperture; 2.0 mm working distance) using Nikon NIS Elements AR software. DyLight 649 was excited by a 640-nm laser. At each position, image stacks of 16 z-slices (4.6667 μm step size) were acquired with an 18.9-μm pinhole size (voxel size = 1.3811 μm^3^ × 1.3811 μm^3^ × 4.6667 μm^3^) and a 1-μs pixel dwell time. An average of two scans was used for each image. Other acquisition parameters (laser intensity and detector gain) were optimized to prevent over-saturation of the detectors.

#### 2.1.11 Image analysis

**FIGURE 5:**
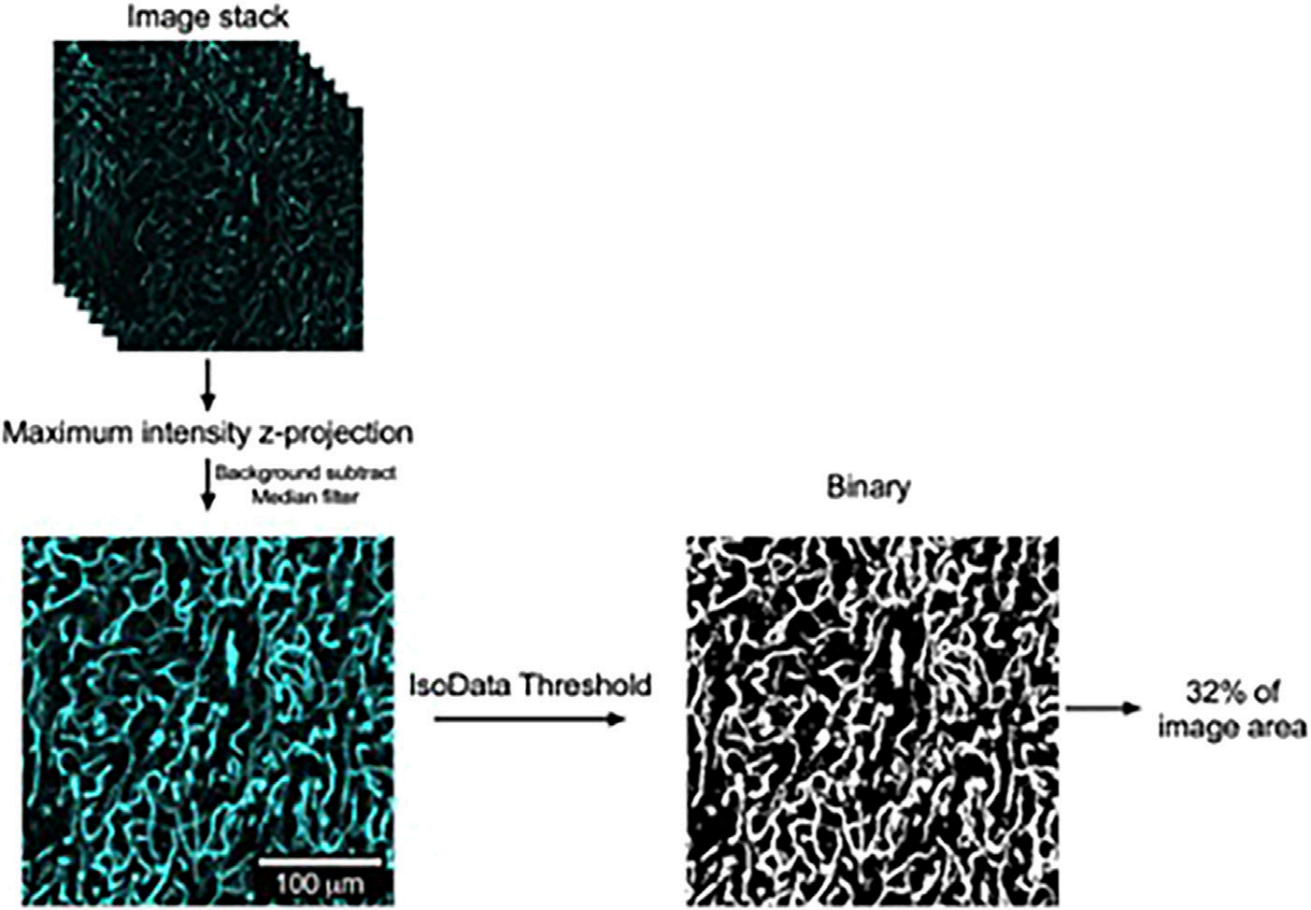
Image analysis to quantify vascular density in lectin-stained aortic PVAT. Schematic representation of image processing used to quantify vascular density in aortic PVAT with examples of image regions of interest used in this process. Confocal image stacks were acquired as stated in the text and opened in FIJI version of ImageJ. Maximum intensity z-projections were then computed. After background subtraction and the application of a median filter, the resultant gray-scale images were converted to binary by the application of an IsoData threshold. The percentage of area occupied by white pixels was then computed as a measure of aortic vascular density, as shown. The contrast and brightness of the maximum intensity z-projection image in this figure were adjusted for display purposes.

Image stacks were opened in FIJI (ImageJ), and maximum-intensity z-projections of the image stacks were computed. The resultant images were then background-subtracted, followed by the application of a median filter. The gray-scale images were then converted to binary images by applying the IsoData threshold. Regions of interest (ROIs, approximately 169 μm × 169 μm) were then randomly applied to three locations in each of the three lobes of PVAT. We then measured the fraction of each ROI occupied by white pixels as a measure of vascular density and took the mean of all ROIs for each animal to allow the comparison of vascular density in the three lobes of PVAT from anterior, middle, and posterior locations along the thoracic aorta by two-way ANOVA (Figure 5).

#### 2.1.12 Statistics

Data are reported as the mean ± SEM of the % area, with n-values representing the number of rats studied (3) and individual values representing the mean of 3–6 sections of aortic PVAT. Data were analyzed using a two-way ANOVA, with the main factors being the three lobes of PVAT (ventral, lateral 1, and lateral 2) and the position along the aorta (anterior, middle, and posterior). p-values <0.05 are designated as statistically significant.

### 2.2 Materials and equipment required

The details of materials needed are as follows (roughly in order of use):

Lectin 649 [LEI, TL, DyLight 649, VectorLabs, DL-1178, lot 2K0717, 1 mg (1 mg/mL)], 31G insulin syringe, 0.9% saline, 18G needle, lab tape, DMEM/F12 media, specimen cup, 4% paraformaldehyde, sterile ethanol wipes, ice, surgical tools, PBS with sodium azide, 10-mL glass scintillation vials, tetrahydrofuran (THF), ultrapure water, 10-mL syringe, sucrose, 15-mL conical tubes, embedding molds, O.C.T., ethanol, 2-methylbutane, dry ice, cryostat, paint brushes, sectioning blades, microscope slides, razor blade, slide tray, slide tox, 70% ethanol, Kimwipes, DAPI 1×, PBS, EZClear Imaging Solution, coverslips, custom slide tray for staining, and pap pen.

### 2.3 Reagents ordering information

**Table:** 

Item	Source	Catalog number	Specification on use
Lectin 649 (*Lycopersicon esculentum* (tomato) (LEL, LT) DyLight 649)	VectorLabs	DL-1178-1	750 ul per rat
31G insulin syringe	Fisher Scientific	50-209-2889	0.31 mm × 8 mm, 30G5/16, 0.5
0.9% sodium chloride	BD (Fisher Scientific)	BD ref 306,546	BD PosiFlush Normal Saline Syringe
Lab tape	Fisher Scientific		
Monobasic sodium phosphate	Fisher	3818-01	
Dibasic sodium phosphate	Fisher	3828-01	
DMEM/F12 media (HEPES, No phenol red)	Fisher Scientific	11039047	
Specimen cup	VWR	60820-102	
Paraformaldehyde	Sigma	158127-500 g	
Sterile ethanol wipe	Fisher Scientific	Ref 5110	
DAPI 4′-6′-diamidino-2-phenylindole-dihydrochloride	Sigma	D8417-1 mg	
Nycodenz	Accurate Chemical	AN1002423, 100 g	
Urea	Sigma	U5378	
Sodium azide	Sigma	S2002	
Bottom–top filtration system	Fisher Scientific	Z370606	Nalgene, pore size 0.2 um
Coverslips	Fisher Scientific	FisherFinest Premium Coverglass, 24x50-1, #125485M	
Slide tray	Ted Pella	2113-B	Plastic slide holder tray with black plastic-hinged cover
Pap pen	Vector Laboratories	H-4000	ImmEdge Pen
O.C.T.	Fisher Scientific	4585	Fisherbrand
Sectioning blades	Stukey		
Microscope slides	Fisher Scientific	12-550-15	Fisherbrand Superfrost Plus Microscope Slides, precleaned, white, 25 mm × 75 mm × 1.0 mm
Razor blades	Fisher Scientific	12-640	Fisherbrand Razor Blades
Slide box	Fisher Scientific		
Kimwipes	Fisher Scientific		
Embedding molds	Polysciences, Inc., 18646A-1	18646A-1	Peel-A-Way Embedding Molds, Square S22
Ethanol, absolute 200 proof	Fisher Scientific		
2-Methylbutane	Fisher Scientific	Q223-07	
15-mL conical tubes	VWR	89039-666	
Tetrahydrofuran	Sigma	186562	
Ultrapure water	HyClone	SH30538.03	
18G needle	BD	Ref 30519	18G × 1 ½ TW (1.2 mm × 40 mm)
10-mL syringe	BD	Ref 302995	
Sucrose	Fisher Scientific	4072-05	

### 2.4 Buffers and solution compositions

1× PBS– 14.4 g Na_2_HPO_4_
     2.4 g KH_2_PO_4_
     2.0 g KCl
     80.0 g NaCl
     2.9 g Na Azide
     900 mL dH_2_O

- Mix until dissolved fully. pH will be 7.4. Adjust the final volume to 1L with dH_2_O.
- Note that PBS can also be purchased from many vendors, although it may lack sodium azide.

- 4% PFA–Mix 20 mL of 0.2 M phosphate buffer with 10 mL of dH_2_O and 10 mL of 16% PFA.
- 16% PFA–Dissolve 16 g of paraformaldehyde with 100 mL of dH_2_O heated to 60°C, add two drops of 10 N NaOH, and continue to stir until fully dissolved. Cool to room temperature, then filter, and store at 4°C.
- 0.2 M Phosphate buffer–6.9 g monobasic sodium phosphate
- 21.3 g dibasic sodium phosphate
- 900 mL dH_2_O

- Mix until fully dissolved and then adjust the final volume to 1 L with dH_2_0. pH will be ∼7.2–7.4.
- To make 0.02 M phosphate buffer, dilute 100 mL of 0.2 M phosphate buffer with 900 mL of dH_2_O (1:10 dilution).

EZClear Imaging Solution–Stir 52.5 g of urea and 31.25 mg of sodium azide with 35 mL of 0.02 M phosphate buffer (pH 7.4) in a 250 mL beaker, heated to 37°C on a hot plate. Once the urea is dissolved fully, slowly add 100 g of Nycodenz to the solution and stir until fully dissolved. Adjust the final volume to 125 mL with 0.02 M of phosphate buffer. Filter using the bottle–top vacuum filtration system and store at room temperature. The refractive index of the EZClear solution is between 1.512 and 1.518.

## 3 Results

**FIGURE 6:**
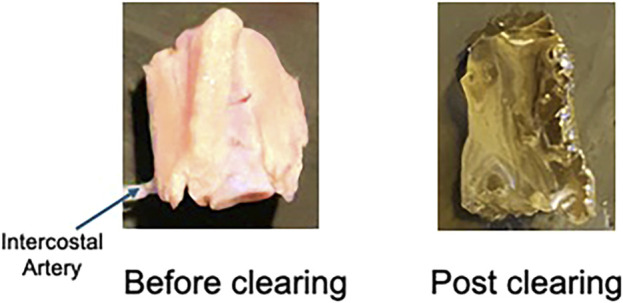
Image of a naïve/uncleared thoracic aortic ring with PVAT (left) and a cleared right (right). Representative of the three male rats used in this study and the 9–12 rings processed.

Thoracic aortic PVAT was successfully cleared using this protocol. Figure 6 shows an image of the thoracic aortic ring before (left) and after (right) the above-outlined protocol. For all three rats, significant color was lost, and translucency was gained in all three sections (anterior, middle, and posterior) taken through the protocol. Tissues did not become completely colorless but rather a light yellow.

**FIGURE 7:**
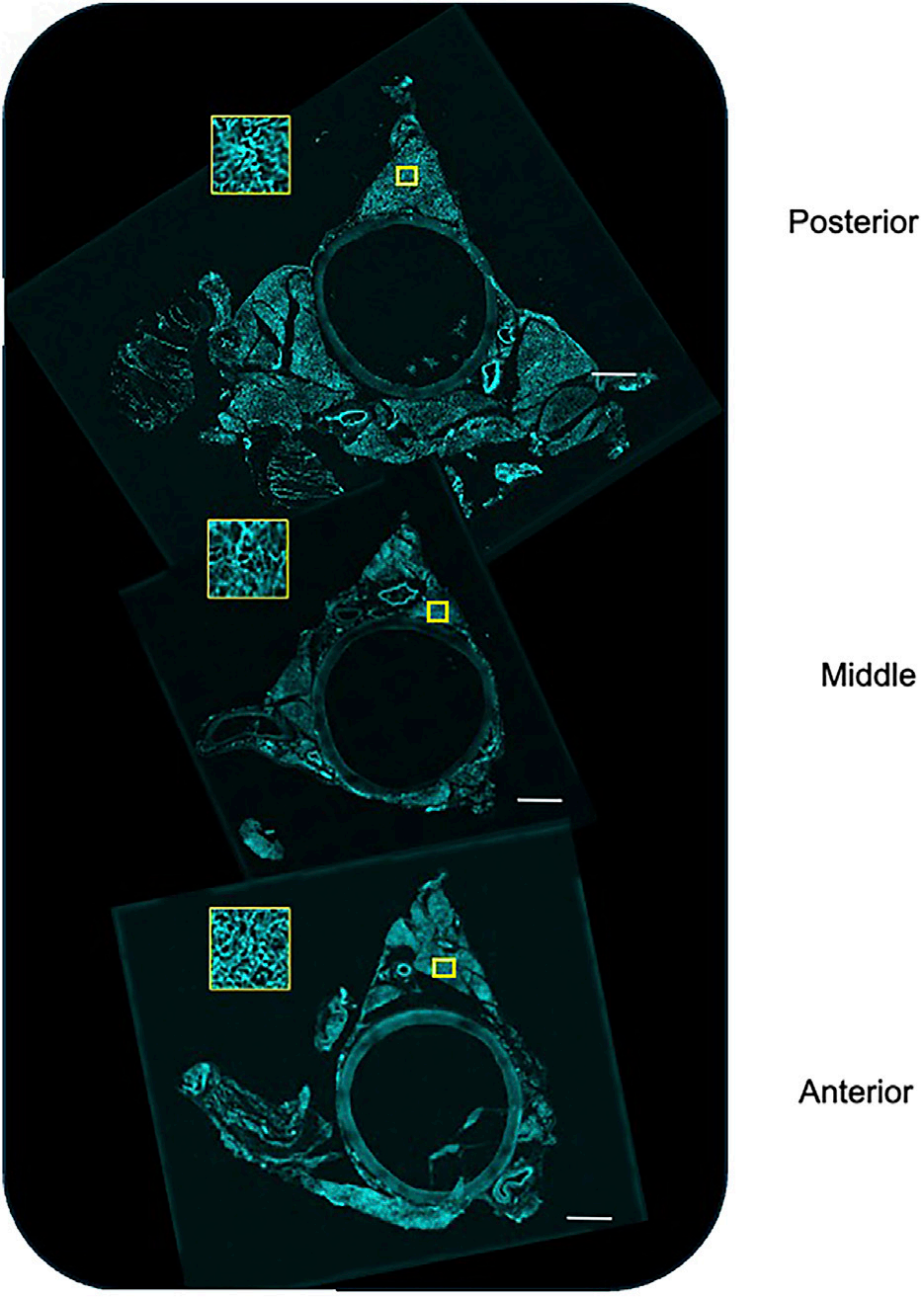
Stitched images of the posterior (near diaphragm), middle, and anterior (near heart) of the whole aorta stained, *in vivo*, with Lectin 649 to mark the vascular endothelium. The yellow box depicts a closer view of the marked region of interest. Representative of the three male rats used in this study.

Figure 7 shows the images of the imaged anterior (near heart), middle, and posterior (near diaphragm) sections of the whole thoracic aorta. The PVAT around the aorta was irregular, with one lobe (ventral) being free, while the other two lobes (lateral) attach the aorta to the spine. Therefore, the removal of these lobes causes fractures/tears within the PVAT that are the apparent raggedness of the lateral lobes. Nonetheless, all PVATs possessed a microvasculature. This was visualized by the cyan color that stained the microvasculature. The yellow boxes show a high magnification of the microvasculature.

**FIGURE 8:**
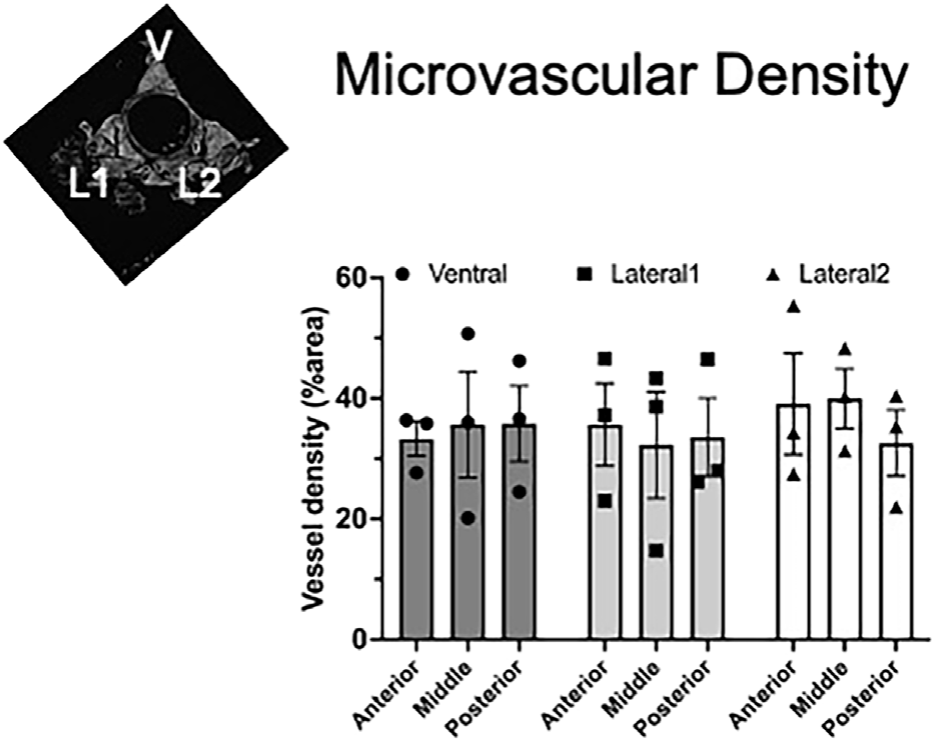
Homogeneous vascular density in aortic PVAT. Inset figure depicts where ventral (V), lateral 1 (L1), and lateral 2 (L2) PVAT lobes are located. Data are presented as the mean ± SEM of % area from three rats, where each data point represents the mean of sequential sections. Two-way ANOVA revealed no significant differences among the lobes or the positions along the aorta (p > 0.82 for all comparisons).

We found that aortic PVAT had a relatively high microvascular density amounting to 32%–35% of the areas analyzed. We found no significant difference in the density among the three lobes or along the length of the aorta (Figure 8).

## 4 Discussion

### 4.1 Clearing of rat PVAT is feasible

The protocol and findings shared in this study demonstrate that a clearing method, adapted from a mouse/brain clearing protocol (Hanscom et al., 2024), can successfully be used in PVAT from the rat. Although we demonstrate the use of this method for visualizing the microcirculation, it would seem possible that other structures within the tissue could also be similarly visualized, depending on the availability of the tool that would well-label the target of interest. In this study, we used Lectin-649 as a marker of vascular endothelium, a well-accepted use of this lectin (Xie et al., 2024).

With this method, the finer structures of the vascular endothelium in PVAT were visualized and shown to be present in all lobes (ventral and lateral) of the anterior, middle, and posterior of the thoracic aortic PVAT. This finding provides an explanation for why so many endothelial cells and, to a lesser extent, smooth muscle cells, were observed in snRNA-seq experiments of the same thoracic aorta PVAT (Thompson et al., 2024).

### 4.2 The knowledge of a present microvasculature in PVAT is important

The hypoxia of tissues, as is observed in adipose tissue with obesity, is thought to occur because of vascular rarefaction (AlZaim et al., 2023). The loss of microvasculature deprives the cells of the adipose tissue of oxygen and nutrients, thereby creating an “unhealthy” tissue that experiences oxidative stress and inflammation, two events that are characteristic of many chronic diseases. The possibility that this could occur in PVAT must be considered a contributing factor to disease.

PVAT microvasculature is important in another way. Specifically, if PVAT has a microvasculature, it could serve as a source for microvascular fragments useful in engineered tissue constructs (Frueh et al., 2017).

### 4.3 Limitations

Prior to giving the lectin *in vivo*, we tested whether incubating the whole PVAT directly in the lectin would allow for the visualization of the microvessels. However, it did not. Sufficient penetration of the whole PVAT structure with the lectin dye could not be achieved, even after overnight incubation. This certainly would have been a simpler approach but would not have provided the knowledge needed.

This protocol also takes quite some time, although it includes many stop points. We do not know whether some of the commercially available clearing machines, which take significantly less time, could be used with PVAT.

We recognize that light sheet microscopy would allow for a deeper, high-resolution 3D reconstruction of the microvasculature in PVAT, including its association with vasa vasorum. We were unable to obtain access to a light microscope for this study (use indicated in Figure 1), but we intend to do so in the future.

### 4.4 Moving forward

With the validation that a microvasculature exists in rat PVAT, the next steps include reconstructing three-dimensional images of PVAT such that microvascular density, tortuosity, and volume can be constructed in the normal state. This knowledge allows for comparison to diseased states, where one could hypothesize that rarefaction of the PVAT microvasculature occurs.

## Data Availability

The original contributions presented in the study are included in the article/supplementary material; further inquiries can be directed to the corresponding author.
